# Single-cell RNA sequencing in endometrial cancer: exploring the epithelial cells and the microenvironment landscape

**DOI:** 10.3389/fimmu.2024.1425212

**Published:** 2024-08-20

**Authors:** Silvia González-Martínez, Belén Pérez-Mies, Javier Cortés, José Palacios

**Affiliations:** ^1^ “Contigo Contra el Cáncer de la Mujer” Foundation, Madrid, Spain; ^2^ Molecular Pathology of Cancer Group, Ramón y Cajal Health Research Institute (IRYCIS), Madrid, Spain; ^3^ Centre for Biomedical Research in Cancer Networks (CIBERONC), Carlos III Health Institute, Madrid, Spain; ^4^ Department of Pathology, Ramón y Cajal University Hospital, Madrid, Spain; ^5^ Faculty of Medicine, University of Alcalá, Madrid, Spain; ^6^ International Breast Cancer Center (IBCC), Pangaea Oncology, Quiron-salud Group, Barcelona, Spain; ^7^ Medica Scientia Innovation Research, Barcelona, Spain; ^8^ Medica Scientia Innovation Research, Ridgewood, NJ, United States; ^9^ Department of Medicine, Faculty of Biomedical and Health Sciences, European University of Madrid, Madrid, Spain; ^10^ IOB Institute of Oncology Madrid, Hospital Beata María Ana de Jesús, Madrid, Spain

**Keywords:** single-cell RNA sequencing, endometrial cancer, tumor microenvironment, immune landscape, cellular heterogeneity

## Abstract

Single-cell RNA sequencing (scRNA-seq) technology has emerged as a powerful tool for dissecting cellular heterogeneity and understanding the intricate biology of diseases, including cancer. Endometrial cancer (EC) stands out as the most prevalent gynecological malignancy in Europe and the second most diagnosed worldwide, yet its cellular complexity remains poorly understood. In this review, we explore the contributions of scRNA-seq studies to shed light on the tumor cells and cellular landscape of EC. We discuss the diverse tumoral and microenvironmental populations identified through scRNA-seq, highlighting the implications for understanding disease progression. Furthermore, we address potential limitations inherent in scRNA-seq studies, such as technical biases and sample size constraints, emphasizing the need for larger-scale research encompassing a broader spectrum of EC histological subtypes. Notably, a significant proportion of scRNA-seq analyses have focused on primary endometrioid carcinoma tumors, underscoring the need to incorporate additional histological and aggressive types to comprehensively capture the heterogeneity of EC. By critically evaluating the current state of scRNA-seq research in EC, this review underscores the importance of advancing towards more comprehensive studies to accelerate our understanding of this complex disease.

## 1 Introduction

**Figure 1:**
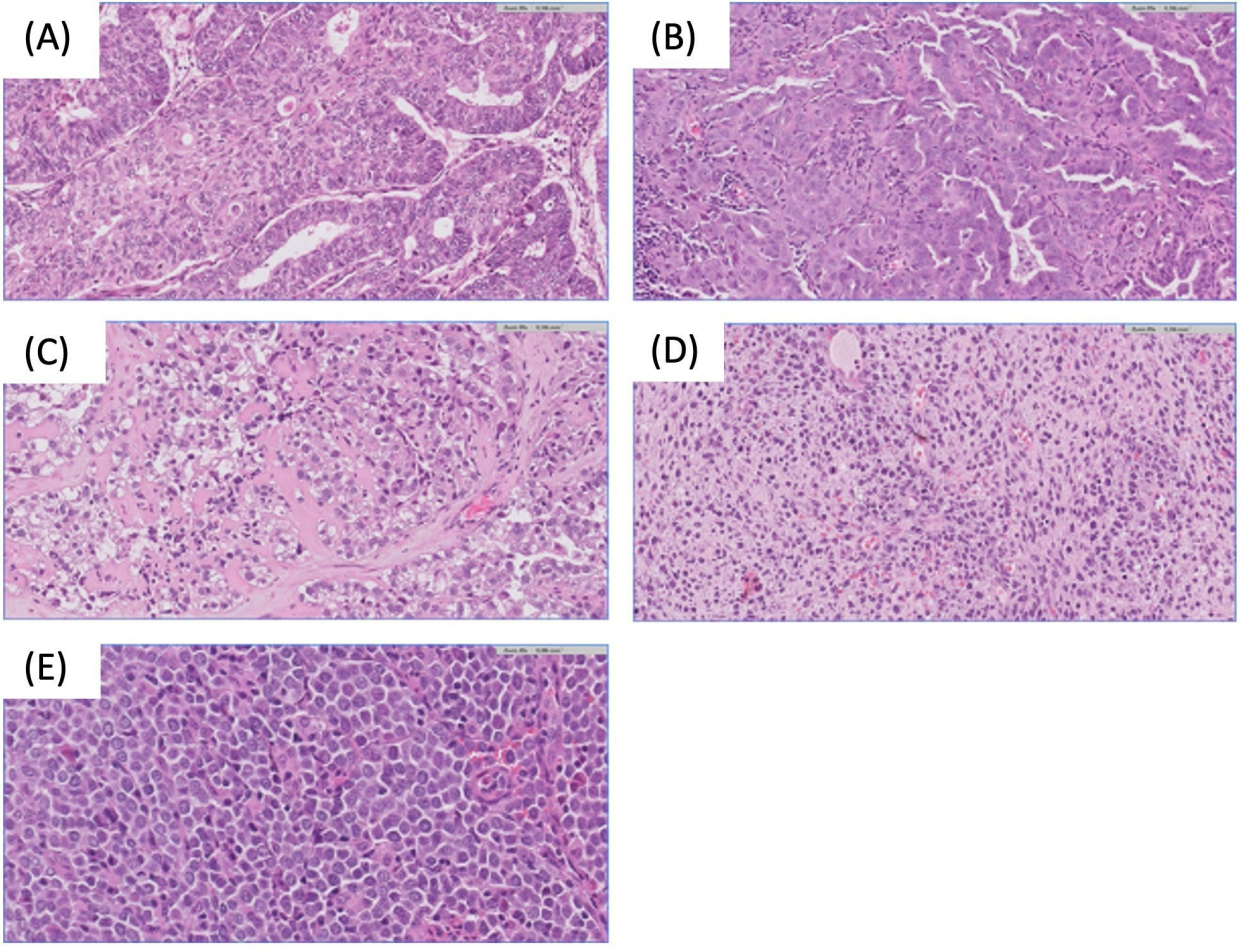
Endometrial carcinoma histological subtypes. **(A)** Endometrioid endometrial carcinoma H&E 20x. **(B)** Serous endometrial carcinoma H&E 20x. **(C)** Clear cell endometrial carcinoma H&E 20x. **(D)** Sarcomatous component of endometrial carcinosarcoma H&E 20x. **(E)** Undifferentiated endometrial carcinoma H&E 40x.

Endometrial cancer (EC) is a tumor that originates in the inner epithelial lining of the uterus and may exhibit aggressive behavior. It is the most common gynecological malignancy in Europe and the second most common gynecological cancer diagnosed worldwide after cervical cancer, with an incidence of 420,368 cases in 2022. Its prevalence and associated mortality are on the rise globally (1, 2). EC often presents early symptoms, leading to approximately 70% of diagnoses at a localized stage and a favorable 5-year survival rate of around 76% (3). However, 20% of patients experience disease recurrence. Despite an initially high 5-year survival rate of up to 80%, relapse is associated with a rapid progression to the terminal stage (3, 4). Histologically, EC is classified according to the World Health Organization (WHO) system. This categorization divides them into specific subgroups, including endometrioid, serous, clear cell, uterine carcinosarcoma, and other less common types (5). (**Figure 1**)

Endometrioid endometrial carcinoma (EEC) is the predominant type, accounting for 70-80% of all cases. EEC is a diverse subgroup ranging from indolent to highly aggressive forms, with grade 3 EEC posing a significant risk of EC-related deaths (6). Uterine serous carcinoma (SC), the second most common subtype (less than 10% of cases), is notably aggressive and often presents with advanced extrauterine disease. Over 90% of SC cases are associated with *TP53* mutations. Endometrial clear cell carcinoma (ECCC) constitutes less than 6% of all cases, and is characterized by aggressive behavior, resulting in a poor prognosis (7). Uterine carcinosarcoma (UCS) is a rare and highly aggressive tumor, representing about 1.5% of cases and is characterized by epithelial to mesenchymal transition. Molecular studies have identified UCS as tumors with characteristics similar to serous tumors. When the sarcomatous component dominates (i.e. constitutes more than 50% of the tumor), this is associated with a poorer prognosis (8).

**Figure 2:**
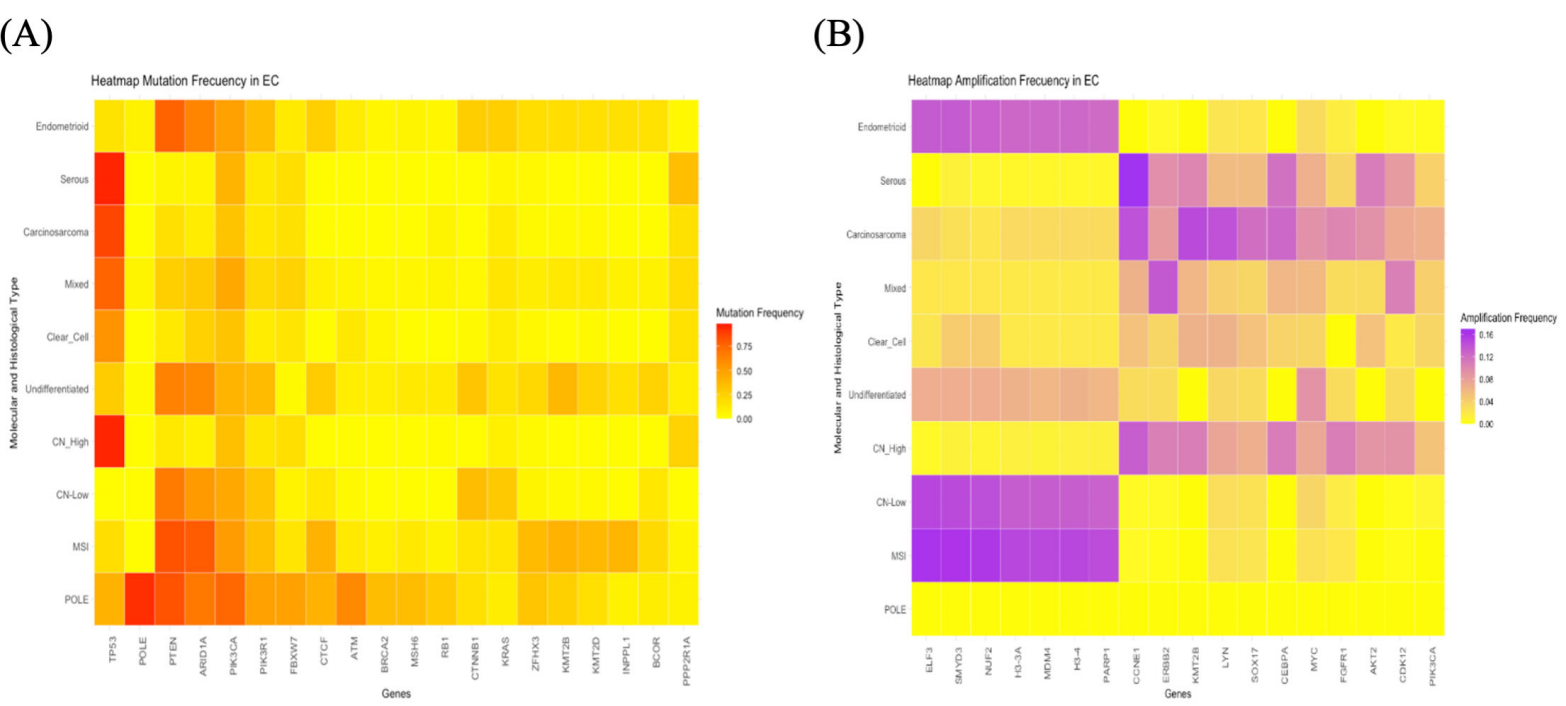
Heatmaps illustrating the most representative molecular alterations in various histological and molecular subtypes of endometrial cancer. **(A)** Heatmap depicting the mutation frequencies of the genes most mutated across different histological and molecular subtypes of endometrial cancer. **(B)** Heatmap illustrating the amplification frequencies of the genes most frequently amplified in the same subtypes.

In 2013, The Cancer Genome Atlas (TCGA) introduced a comprehensive classification system for ECs, dividing them into four molecular categories based on mutational load and copy number alterations identified through genomic and transcriptomic bulk sequencing, as structural variants in any gene did not exceed 1% (4). These categories are as follows: POLE ultramutated (7%), microsatellite instability (28%), somatic copy-number alteration low (39%), and somatic copy-number high (26%). Each category is linked to a distinct prognosis, with POLE ultramutated cases generally associated with the most favorable outcomes and somatic copy-number high cases with the poorest outcome. Additionally, each category presents different potential therapeutic targets (4). This classification entails certain technical challenges and is poorly reproducible outside of the research setting. Therefore, in an effort to devise a tool that could be adapted for daily clinical practice, a classification based on surrogate markers was developed, consisting of the following groups: POLE, which includes tumors with mutations in the exonuclease domain of the POLE gene; mismatch repair deficiency (MMRd), for tumors with loss of expression of mismatch repair pathway proteins; p53 abnormal, for tumors with altered p53 expression; and non-specific molecular profile, for the remaining tumors, which do not exhibit a specific molecular alteration (9). Recently, in the study by Weigelt et al. (10), the largest cohort of EC cases to date was sequenced, comprising 1,882 cases. **Figure 2** illustrates the most frequent mutations (A) and amplifications (B) in each subtype of EC, as visualized in the heatmaps generated from these data.

Lemetre et al. (11), analyzing bulk RNAseq data, obtained results that expanded on the observations that EECs and SCs are distinct. They observed that PI3K signaling, and DNA mismatch repair processes may play a role in the clinical behavior of EECs, as opposed to Wnt signaling (*CTNNB1*) and apoptosis (*CASP3*)-related processes in SCs. Additionally, *MSH6* expression levels may be associated with outcome in ECs as a group; cases with low *MSH6* expression have a more favorable clinical behavior. This finding was recently confirmed by Zhou et al. (12), who additionally observed that the low *MSH6* expression group had a higher immune score, more active immune infiltration, and higher immune checkpoint (IC) expression, resulting in better responsiveness to immune checkpoint inhibitors (ICI) treatment. At the protein level, Dou et al. (13) demonstrated that distinct EC subtypes can be reliably distinguished by their patterns. They identified multiple gene products that are highly expressed in the CNV-high subset of ECs, which includes almost all SCs and many of the high-grade ECs profiled. Of these gene products, *CDK12* and *SMARCA4* can be targeted by FDA-approved drugs.

EC is heterogeneous in it behavior, not only due to differences in the epithelial cells but also by the varied proportions and function of different cell types in the tumor microenvironment (TME) (14). EC TME comprises supportive stromal cells, predominantly cancer-associated fibroblasts (CAFs), immune cells, endothelial cells, and extracellular matrix (ECM) molecules. It has been suggested that these components play a crucial role in tumor progression and metastasis by modulating some processes, such as the immune response and the epithelial-mesenchymal transition (EMT). Certainly, EC is identified as an immunogenetic disease, and extensive research emphasizes the critical role of the tumor-immunosuppressive microenvironment in cancer advancement. Leukocytes, notably tumor-associated macrophages (TAMs), as well as fibroblasts and myofibroblasts, assume a pivotal function in the malignant transition from hyperplasia to EC (15, 16). Contemporary investigations into the TME of EC delve into mechanisms of immune evasion and explore immunotherapeutic approaches, with a primary focus on ICI tailored for EC (17).

**Figure 3:**
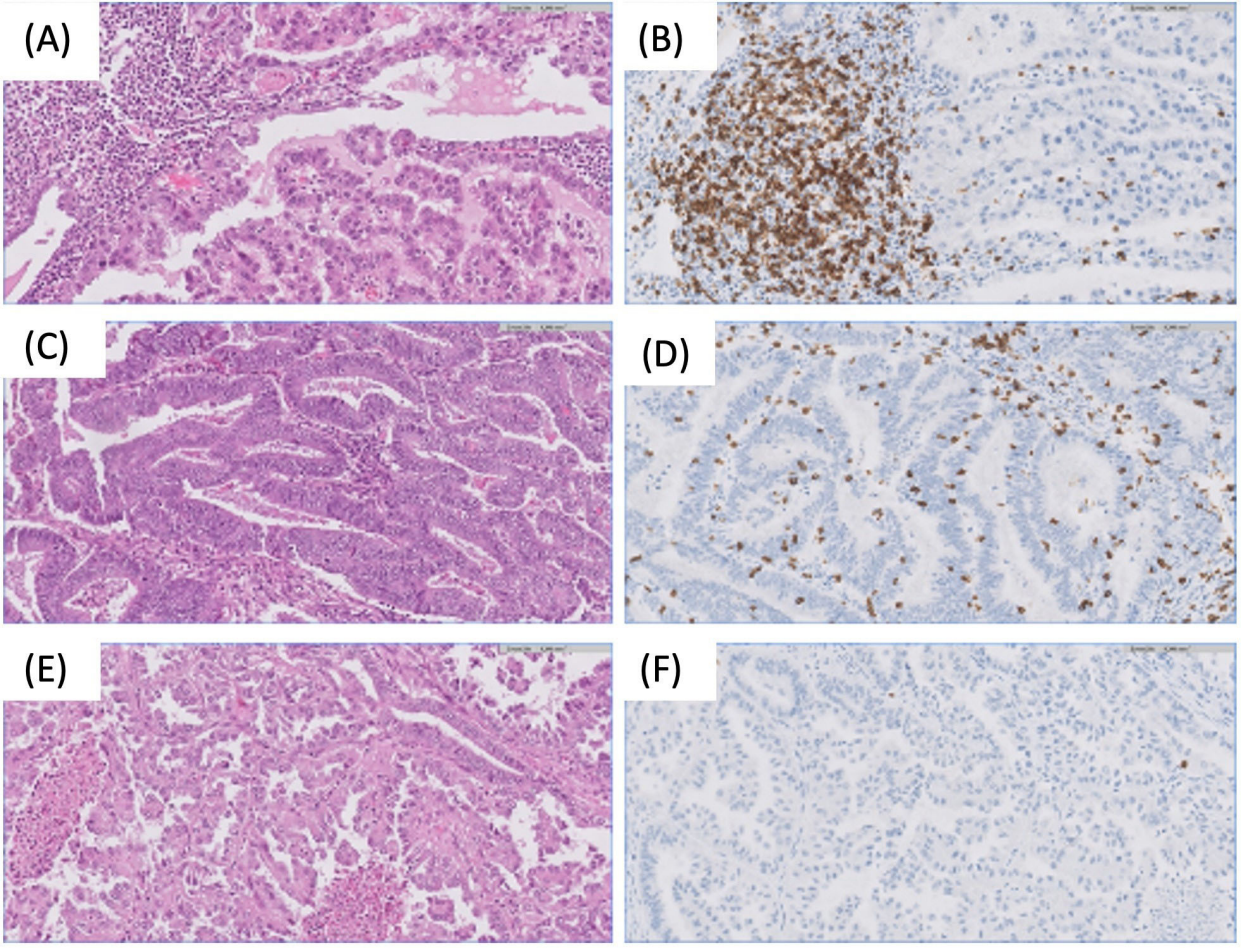
Examples of different microenvironment across endometrial tumors. **(A, B)** Endometrial clear cell carcinoma with dense stromal immune infiltration, predominately formed by CD8+ lymphocytes. H&E and CD8 20x. **(C, D)** Endometrial endometrioid carcinoma with high number of intraepithelial CD8+ lymphocytes. H&E and CD8 20x. **(E, F)** Endometrial serous carcinoma without immune response. Note the absence of CD8 lymphocytes. H&E and CD8 20x.

On the other hand, cells associated with a favorable prognosis have also been described in the EC TME. There is a substantial presence of tumor-infiltrating CD8+ T lymphocytes within cancer cell nests, particularly abundant in microsatellite instability tumors. POLE ultramutated tumors, also exhibit significant tumor-infiltrating lymphocytes (TILs) infiltration, marked by the overexpression of genes associated with the cytotoxic functions of TILs (18) (**Figure 3**). However, the anti-tumor response can vary. In fact, Chow et al. (19) in a phase 2 clinical trial of the PD-1 inhibitor pembrolizumab in patients with MMRd found contrasting modes of anti-tumor immunity for mutational vs. epigenetic MMRd cancers. While effector CD8+ T cells correlated with regression of mutational MMRd tumors, activated CD16+ NK cells were associated with ICI-responsive epigenetic MMRd tumors. More recently, Guo et al. (20) also confirmed the heterogeneous TME status within MMRd ECs. They demonstrated that these ECs can be stratified based on potential biomarkers such as HLA class I, *DNMT3A*, and CD8 in pathological settings to improve ICI therapeutic efficacy in this subset of patients. These data highlight the interplay between tumor-intrinsic and extrinsic factors that influence ICI response.

Furthermore, the results by Zhang et al. (21) highlight the intricate interplay among genomic alterations, immune cell infiltration, and the TME in EC. Their study suggested that specific immune cell populations, rather than the overall immune landscape, were associated with the prognosis of patients with EC. A Tumor infiltrating Immune Cell Score (TICS) was constructed using CD8+ T cells, resting memory CD4+ T cells, activated NK and activated DCs, and classified patients as low-, moderate- and high-risk subgroups. The low-risk subgroup identified by TICS was associated with microsatellite instability or POLE subtype, higher TMB score, and activation of various immune response signaling. On the other hand, the high-risk subgroup was significantly correlated with higher fraction of *TP53* subtype, activation of EMT, hypoxia and *KRAS* signaling. Therefore, gaining a deeper understanding of the heterogeneity and intratumor crosstalk among distinct cells in the TME, including tumor cells, holds the potential to identify more effective therapeutic targets for EC.

**Figure 4:**
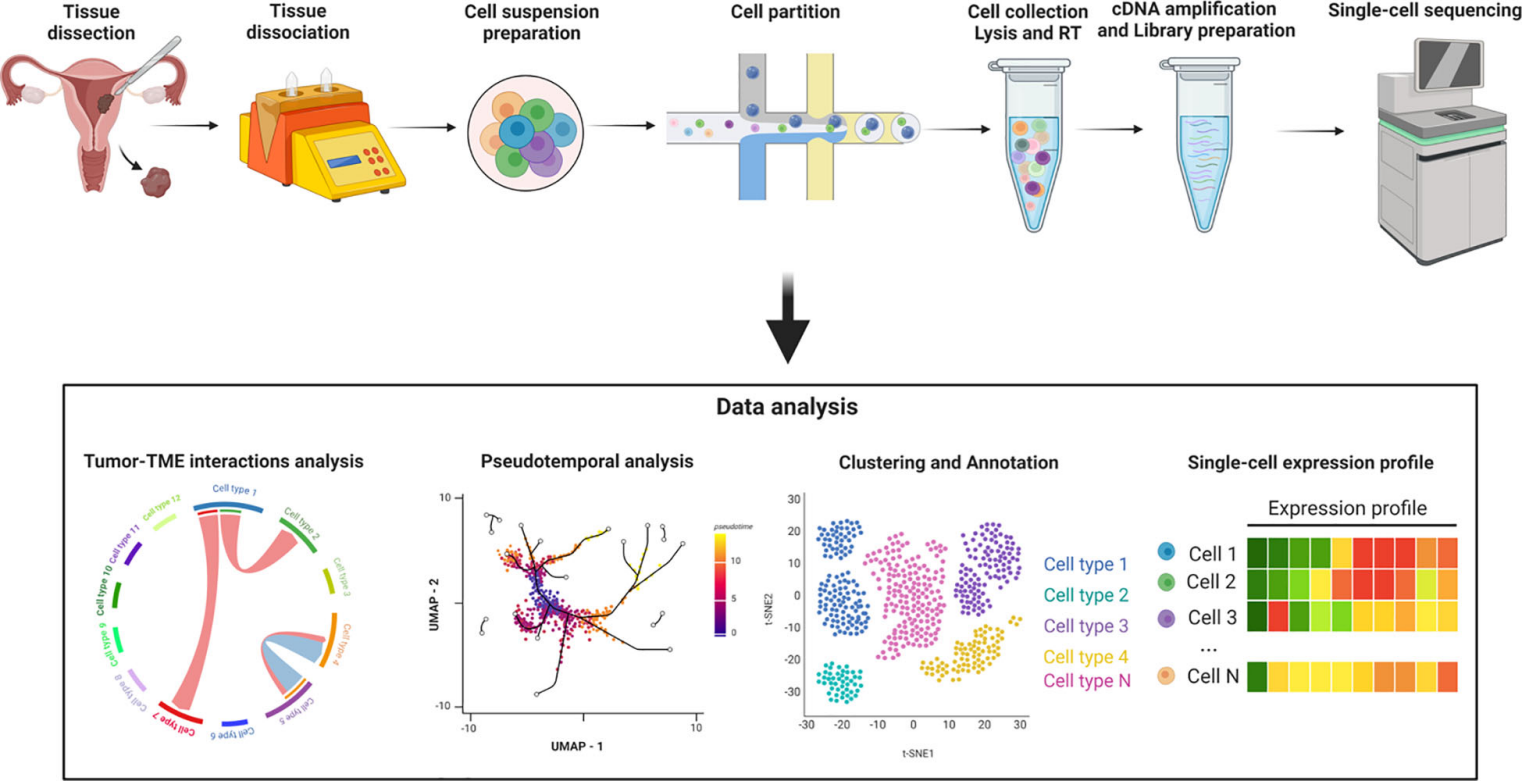
Workflow of the single-cell RNA sequencing process. Created with BioRender.com.

Single-cell RNA sequencing (scRNA-Seq), as an emerging sequencing technology, serves as a robust tool for characterizing cell subpopulation classification and cellular heterogeneity. It enables high-throughput and multidimensional analysis of individual cells, overcoming the limitations of traditional sequencing methods that only detect the average transcript level of cell populations (22). Additional techniques, such as single-cell assay for transposase-accessible chromatin sequencing (scATAC-seq) and ChIP sequencing (scChIP-seq), explore chromatin accessibility and transcriptional factor regulation at the epigenetic level (23, 24). The advancement of integrated tools for single-cell studies not only reveals tumor heterogeneity but also facilitates the exploration of cell-cell interactions and improves the resolution of rare cell populations, contributing to a more profound understanding of the nature of cellular malignancy (25, 26). (**Figure 4**)

This review aims to summarize the latest information obtained through scRNA-seq from EC to better understand of the intricate dynamic relationships between tumor cells and TME cells.

## 2 Results

### 2.1 Single-cell RNA sequencing studies in endometrial cancer

**Figure 5:**
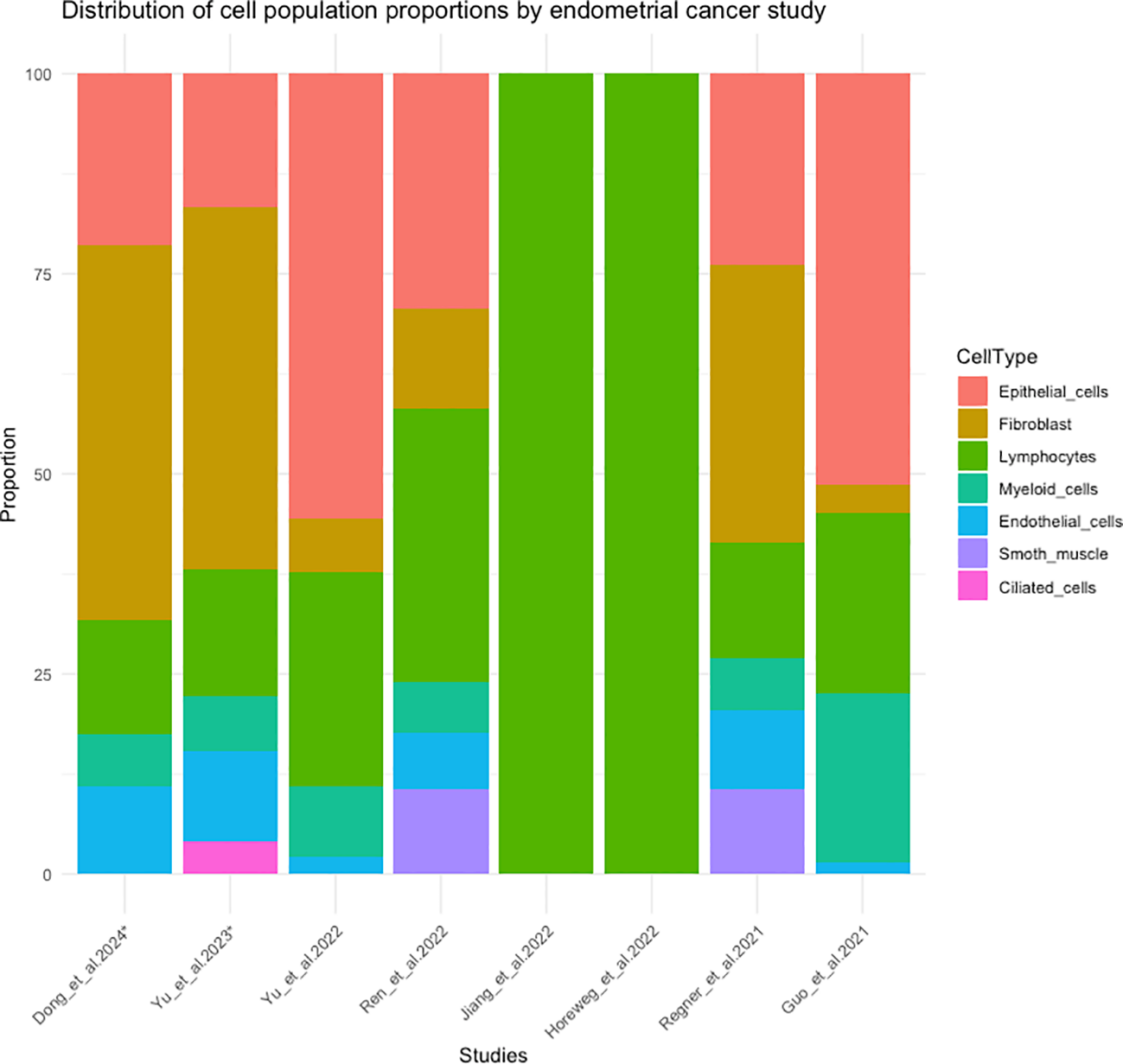
Distribution of cell population proportions in 8 studies of scRNA-seq in endometrial cancer. (*) The cases correspond to the 5 endometrioid endometrial cancer cases from the study by Regner et al. (29).

There are limited studies analyzing EC using scRNA-Seq (27–35), encompassing a total of 29 ECs (28 primary tumors and one ovarian metastasis), with a varying number of cells per case, ranging from 2,500 to 13,207 cells (including different populations) or ≈250 cells including only B lymphocytes (see **Supplementary Table 1**). Additionally, the proportion of each cell population varied across studies. Epithelial cells were the predominant population in the studies by Yu et al. (27) and Guo et al. (28), while fibroblasts were predominant in the samples analyzed by Regner et al. (29). Consequently, fibroblasts were also predominant in the studies by Dong et al., Yu et al. and Wu et al. (30, 32, 34), which analyzed 5 EEC of the Regner et al. (29) study. Lymphocytes population was the predominant one in Ren et al. (33) study and the only one in the studies by Jiang et al. and Horeweg et al. (31, 35), who included only T cells and B cells respectively. In **Figure 5**, the distribution of cell population proportions from 8 EC studies can be observed (27–29, 31–35). In Wu et al. study (30), this data was not available, but they conducted their analysis using the same 5 EECs as in the Dong et al. and Yu et al. study (32, 34).

Regarding clinicopathological features, the median age in the studies where this data was available was 70 (29, 30, 32, 34), 50 (33), 62.5 (27) and 55 (28) years old. In relation to histological type, 14 cases were endometrioid, 1 case was serous, and 6 cases were not specified. Concerning molecular phenotype, 4 were identified as mismatch repair deficient, 8 cases had a non-specific molecular profile, and 16 cases were not specified. In terms of stage, 13 cases were stage I, 1 was case stage II, 1 case was stage III (corresponding to the ovarian metastasis of EC), and 14 cases were not specified. Regarding histological grade, 12 cases were grade 1, 2 were grade 2, 2 were grade 3, and 13 were not specified. (See **Supplementary Table 2**).

The quality filters for expressing genes, UMI counts, and mitochondrial fraction applied in each study varied and may impact the total yield for scRNA-seq studies (see **Supplementary Table 3**). Additionally, doublets in the matrix were analyzed in only 2 of the studies (27, 29), and batch effects were corrected in 4 studies (31–34) (see **Supplementary Table 4**).

### 2.2 Epithelial cell heterogeneity in endometrial cancer

Epithelial cells play a pivotal role in understanding the complex landscape of EC and scRNA-seq studies have unraveled unique gene expression patterns within different groups of epithelial cells (27–29, 32). This heterogeneity within the malignant epithelial cell population highlights the diverse roles these cells may assume in the progression of EC, offering valuable insights into the underlying cellular dynamics of this condition.

García-Alonso (36), using single-cell transcriptomics data of normal endometrium tissues as a reference to deconvolute RNA bulk data of TCGA ECs, demonstrated that endometrial carcinomas exhibit a less differentiated epithelial phenotype compared to normal endometrium. Endometrial adenocarcinomas displayed two main signatures that were characteristic of the proliferative phase endometrium, SOX9+LGR5+ and SOX9+LGR5−, indicating differences in pathogenesis and disease progression. SOX9+LGR5+ was the dominant signature in serous endometrial (63%) and some endometrioid adenocarcinomas (24%) and was positively associated with the ‘Copy-Number high’ molecular subtype from TCGA, as well as the clinically more aggressive stage III and IV adenocarcinomas. SOX9+LGR5− was dominant in endometrioids (33%) and absent in serious cases. Furthermore, less than 10% of EC expressed signals of glandular and ciliated cells, characterized by expression of *SCGB2A2, PAEP* or *PIFO.*

Yu et al. (32) and Dong et al. (34) conducted a reanalysis of the 5 cases of EEC originally studied by Regner et al. (29). Yu et al. (32) highlighting that batch effects were not corrected in the previous study. The malignant epithelial cells were further classified into five clusters (Ep.0, Ep.1, Ep.2, Ep.3, Ep.4). The cluster 0 was very different from the other four clusters. Clusters 1 and 2, as well as clusters 3 and 4 were similar. Furthermore, Ep.1 and Ep.2 were more malignant than other subclusters (see **Supplementary Table 5**). Additionally, they demonstrated that, in their analysis of cell communication, the epithelial cells emitted the strongest signals, with the top pathway being the MK pathway. They found that cells mainly communicated with each other through Ligand-Receptor (L-R) pairs of MDK–NCL in the MK signaling pathway. Midkine (MK, MDK) was primarily expressed in epithelial cells and ciliated cells, while nucleolin (NCL) was expressed in all other cells. This finding underscores the potential role of MDK in the complex dynamics of EEC, suggesting its potential impact on the TME and disease progression. In the study by Dong et al. (34), the epithelial cells were analyzed to understand the relationship between the heterogeneity of EC epithelial cells and the activation of the estrogen signaling pathway. They observed a cluster with high expression of *MUC1* and *ELF3*, estrogen-associated genes that influence cancer cell proliferation, migration, and invasion (see **Supplementary Table 5**).

Yu et al. (27) included 3 moderately differentiated EC and 1 well differentiated EC, and identified 5 classes of tumoral epithelial cells (C1, C2, C3, C5, C6). Each class displayed a specific gene expression pattern and distinct functions: the PPAR signaling pathway for C1, glycosaminoglycan biosynthesis heparan sulfate for C2, DNA replication and the P53 signaling pathway for C3, the calcium signaling pathway for C4, the intestinal immune network for IgA production for C5, and cytokine-cytokine receptor interaction for C6 (see **Supplementary Table 5**).

Guo et al. (28) compared gene expression differences in epithelial cells between 5 EEC samples and their corresponding normal tissues of 3 of the cases. They identified 227 differentially expressed genes, with gene set enrichment analyses highlighting the upregulation of genes associated with cancer-related functions in tumor samples, such as epithelial cell proliferation and enhanced RNA polymerase II (Pol II) function, indicative of a malignant state. Conversely, genes expressed at higher levels in normal tissue were linked to the negative regulation of proteolysis and its enzymes, suggesting a potential role of proteolytic enzymes in the tumor microenvironment and their involvement in tumor progression. Three epithelial subtypes (stem-like cells, secretory glandular cells, and ciliated cells) were identified in both endometrial tumor tissue and normal endometrial tissue. However, a notable proportion of cells in tumor samples could not be classified into these subtypes. Ciliated cells exhibited high expression levels of markers related to motile cilia and ciliogenesis, with pathways associated with cilia organization, assembly, and movement being prominent. Secretory glandular cells showed elevated expression of genes related to epithelial cell development and differentiation, along with pathways related to extracellular matrix, cell adhesion molecules, and leukocyte migration, indicating potential interactions with surrounding stromal cells. Stem-like cells, lacking specific marker genes, displayed elevated expression of ribosomal genes, suggesting the presence of stem/progenitor cells (see **Supplementary Table 5**). Additionally, their pseudo-time analysis unveiled a relative developmental sequence, starting with stem-like cells, followed by secretory glandular cells, and culminating with ciliated cells.

In the study by Ren et al. (33), the evolution of epithelial cells during the development of EEC was examined across different pathological stages. This study included 5 samples of EEC, 5 samples of atypical endometrial hyperplasia (AEH), and 5 samples of normal endometrial tissue. They observed two independent trajectories in epithelial cells and stromal fibroblasts. Additionally, the low expression of mesenchymal-epithelial transition (MET) regulators in stromal fibroblasts suggested that a lineage trajectory of MET may not be valid in EC. They demonstrated that EEC originates from unciliated glandular epithelial cells, and the emergence of LCN2+/SAA1/2+ cells was a feature of endometrial tumorigenesis. Furthermore, by studying the proportion of cell populations, they found that the proportion of epithelial cells increased in AEH and further expanded in EEC, while the proportion of stromal fibroblasts decreased. This insight provides crucial information about the dynamic evolution of endometrial epithelial cells in the context of cancer.

The study of Regner et al. (29) with 5 EEC and 1 ES, revealed that malignant cells not only acquire previously unannotated regulatory elements, driving hallmark cancer pathways, but also display significant variability in chromatin accessibility linked to transcriptional output within the same patients. This emphasizes the importance of intratumoral heterogeneity in understanding EC. They identified three clear examples of cancer-specific distal regulatory elements (dREs) that explain upregulated gene expression in malignant populations relative to normal cell populations in the EEC cohort. For example, there was increased *IMPA2* expression in the malignant fraction of the EEC cohort and increased chromatin accessibility of a cancer-specific dRE within the IMAP2 locus. Similarly, increased *SOX9* and *CD24* expressions in EEC were correlated with three cancer-specific differentially dREs in the malignant fraction.

The exploration of epithelial cells in EC through scRNA-Seq has provided a nuanced understanding of the intricate heterogeneity within the malignant epithelial cell population. The diverse roles assumed by these cells in the progression of EC underscore the complexity of this condition.

### 2.3 Cancer-associated fibroblast heterogeneity in endometrial cancer

Among the multiple components of the TME, CAFs have gained attention for their strong correlation with tumor progression. CAFs are widely recognized for their considerable heterogeneity, which is particularly evident in the substantial subpopulation of CAFs (37).

**Figure 6:**
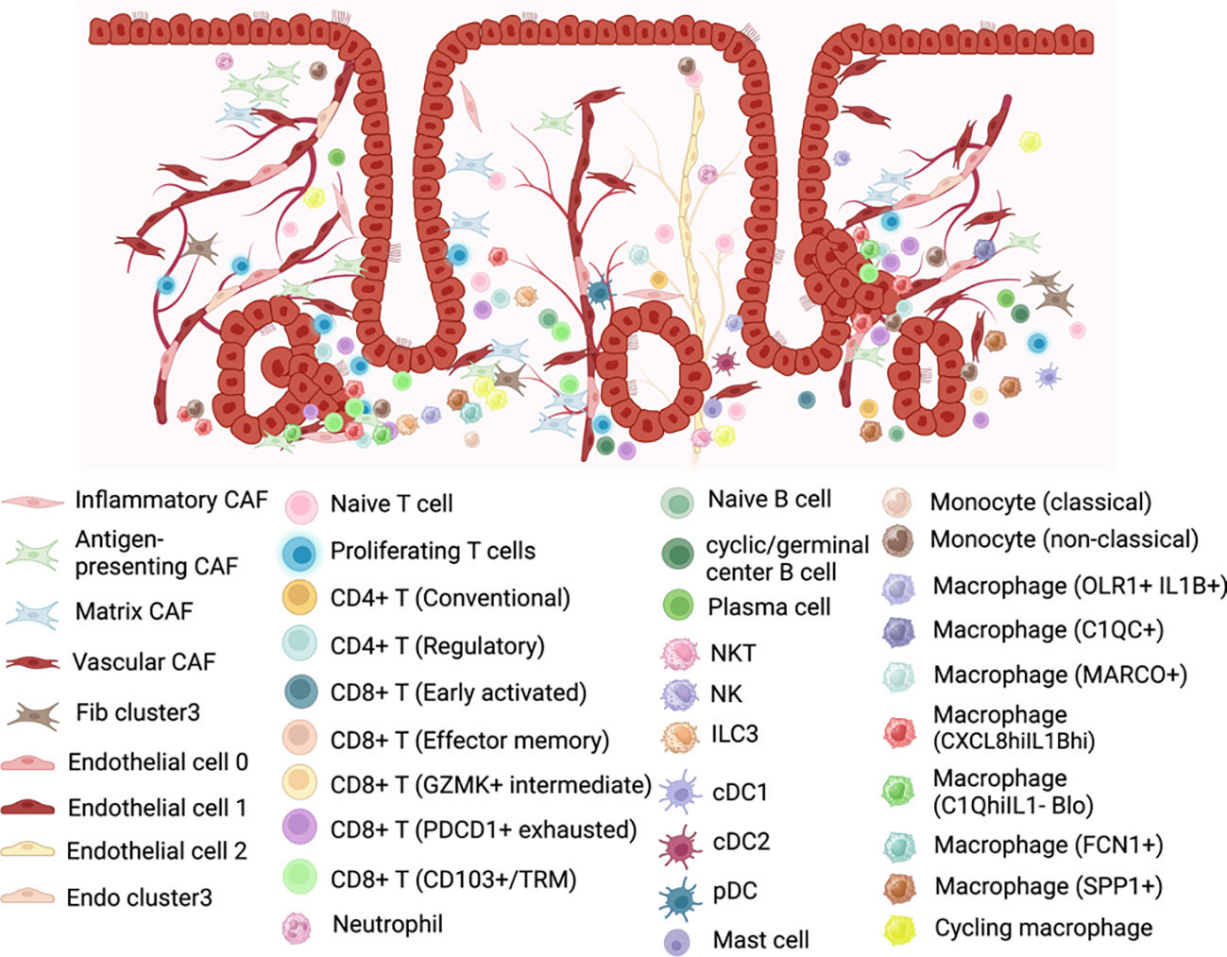
Representation of the cell populations in the endometrial cancer tumor microenvironment. Created with BioRender.com.

To further understand the complexity heterogeneity of fibroblast, Yu et al. (27) classified CAFs from 4 tumors into 4 subclusters: inflammatory CAF population, antigen-presenting CAFs, matrix CAFs, and vascular CAFs. Each subcluster displayed an exclusive expression pattern (see **Supplementary Table 6**; **Figure 6**), implying unique functions within the tumor ecosystem. Comparative analysis of genes between CAFs and fibroblasts in normal endometrium identified up-regulated genes in CAFs that were enriched for processes such as extracellular matrix organization, response to wounding, angiogenesis, and antigen processing and presentation, highlighting distinct characteristics of CAFs. They observed that interactions between malignant and stromal cells, particularly CAFs, were more frequent than those among malignant cells alone. This frequent communication between CAFs and malignant cells was found to facilitate the progression of EC. Notably, vascular CAFs were associated with a poorer prognosis, as evidenced by survival analysis using public endometrial EC data from TCGA.

Contrarily, Ren et al. (33) found that stromal fibroblasts of the endometrium from different pathological stages displayed high similarity to those in normal endometrium. The CNVs and heterogeneity of cell clusters in stromal fibroblasts showed insignificant transcriptome variations among normal, AEH, and EEC samples. However, to confirm the importance of the stromal fibroblast niche for the growth of epithelial tumor cells and to study the differences between stromal fibroblasts from normal endometrium and CAFs from EEC, they performed a co-culture assay using EEC organoids. Stromal fibroblasts from both normal and cancer donors promoted the upregulation of stemness-related genes (*ALDH1, AXIN2, CD133, MYC*, and *SOX9*) in EEC organoids. However, CAFs exhibited a stronger up-regulating effect on the expression of stemness genes in EEC organoids, suggesting that the supportive effect of stromal fibroblasts could be enhanced in the TME.

In the study by Dong et al. (34), fibroblasts were examined to explore the link between the heterogeneity of EC fibroblasts and the activation of the estrogen signaling pathway. They identified 7 clusters, among which Fib cluster3 showed heightened early activation of the estrogen response, with elevated expression of estrogen-related genes compared to other clusters. Furthermore, Gene Ontology (GO) enrichment analysis revealed that genes in Fib cluster3 were predominantly involved in regulating cell proliferation and autophagy signaling pathways (see **Supplementary Table 6**).

### 2.4 Endothelial cells heterogeneity in endometrial cancer

In the study by Yu et al. (32), a total of 3,736 endothelial cells were identified and re-clustered into three distinct clusters (En.0, En.1, En.2). Based on their marker genes, two clusters were characterized as blood endothelial cells (En.0, En.1), while one cluster was designated as lymphatic endothelial cells (En.2). Subsequent analysis revealed that selected genes associated with angiogenesis exhibited high expression levels in the clusters of blood endothelial cells. Pathway analysis further highlighted significant phenotypic diversity among these three endothelial cell clusters (see **Supplementary Table 6** and **Figure 6**). The cell interaction analyses revealed that En.0 received more signals and, along with Ep. 1 and Ep. 2, participated in more pathways compared to other clusters. Additionally, En.0 may be influenced by Ep. 1 and Ep. 2, potentially acquiring a malignant phenotype through MK pathway by MDL-NCL signal. Moreover, they demonstrated by survival analysis with the marker genes that *HSPA1B, TFF3* and *LAMA4* were associated with EC survival.

Including the same cells as Yu et al. (32), Dong et al. (34) proceeded with their analysis with the aim to study the response of endothelial cells to estrogen in EC. They clustered endothelial cells into four subpopulations, including the cluster “Endo cluster3”. Most estrogen-related genes were significantly highly expressed in this cluster (see **Supplementary Table 6**), indicating a higher level of early activation of Endo cluster3 in response to estrogen. Furthermore, GO analysis showed that Endo cluster3 was mainly enriched in pathways related to cell proliferation, migration, positive regulation of cell activation, and epithelial-to-mesenchymal transition.

### 2.5 Immune cells in endometrial cancer

#### 2.5.1 The heterogeneity of lymphocytes

##### 2.5.1.1 NK and T cells

Jiang et al. (35) focused their study on CD45+ lymphocytes from three EC samples. They found that T cells and NK cells comprised the major proportions of EC TILs. They defined three main subsets of NK cells: “NK1”, “NK2”, and “NK3”, which are likely to possess diverse anti-tumor functions. The “NK1” and “NK3” subsets corresponded to the canonical peripheral human NK cell subsets, whereas “NK2” represented a noncanonical population. NK1 cells were identified as likely circulatory NK cells with the strongest cytotoxicity. NK3 cells exhibited strong tissue residency characteristics. The novel NK2 cells had high expression levels of both CD56 and CD16, showing generally intermediate expression levels of inhibitory and activating receptors, and the weakest expression of effector molecules such as granzymes and perforin. The results indicate that NK2 cells might be in a phenotypically intermediate stage of the maturation trajectory from NK1 cells to NK3 cells. However, this hypothesis does not agree with the pseudotime analysis, which suggests that NK2 cells might have a distinct origin from NK1 and NK3 cells.

Yu et al. (27), in their comprehensive analysis of four ECs, observed significant heterogeneity in immune cell subtypes, both within tumors and across individuals. T cells accounted for 23.8% of the total cell population. Their detailed classification revealed four distinct categories: cytotoxic CD8+ T cells, experienced T cells, regulatory T cells, and proliferation T cells (see **Supplementary Table 7** and **Figure 6**). The interaction between epithelial cells and T cells was identified as a key promoter of an immunosuppressive microenvironment in EC, with malignant epithelial cells closely associated with proliferative T cells, promoting regulatory CD4+ and exhausted CD8+ T cell populations. Notably, Ren et al. (33) observed an enrichment of FOXP3+ CD4 regulatory T lymphocytes (Treg) in EEC samples, which are related to immunosuppression, suggesting a potential mechanism of immune escape in EEC.

Guo et al. (28) categorized the lymphocyte repertoire into two main groups: conventional T cells (including conventional CD4+ T cells, regulatory T cells, and CD8+ T cells) and innate-like lymphoid cells (comprising NKT cells, NK cells, and type 3 innate lymphoid cells (ILC3)) (see **Supplementary Table 7** and **Figure 6**). Three subtypes of tumor-infiltrating lymphocytes (CD8+ T cells, regulatory T cells, and ILC3) were correlated with increased overall survival.

When comparing lymphocytes from normal endometrium and EC, Guo et al. (28) observed that early activated CD8+ T cells in normal endometrial tissue expressed *IL7R*, while in tumors, they expressed *XCL1*, with low expression of activated markers such as *HLA-DR*. Effector memory CD8+ T cells in normal tissue were characterized by *GZMH* expression, while GZMK+ CD8+ T cells in tumors represented an intermediate state between effector and exhausted T cells, displaying high expression of activated markers like effector cells but sharing some common genes with exhausted cells, including *PDCD1*. A detailed comparison of exhaustion gene sets in overall normal endometrium and EC revealed higher “exhaustion scores” in CD8+ T cells in tumors, shedding light on the intricate dynamics of the immune landscape in EC and providing valuable insights for further research and potential therapeutic interventions.

Wu et al. (30) observed increased naive T cells, CD8+ T cells, and Treg cells in EC samples compared to normal endometrium, suggesting that CD8+ T cells may play a central role in antitumor immunity in EC. In contrast, Ren et al. (33) observed that the proportions of cytotoxic CD8+ T cell and naive CD8+ T cell were significantly reduced in EEC compared to normal samples. The rise in Treg cells, known for their negative regulatory role in immune regulation, also indicates the complexity of the TME in EC. Jiang et al. (35) focused on the significant role of CD8+ T cells, centering their analysis on the predominant subgroup of CD103+ CD8+ T cells, classified as tissue-resident memory T (TRM) cells. They identified three distinct TRM cell subsets, which expressed common markers like CD69, inhibiting cell egress from tissues. These subsets were primarily differentiated by their expression of IC, classifying them as “IC-low,” “IC-intermediate,” and “IC-high.” The three subsets also differed in the expression of other functional molecules. The “IC-low” and “IC-intermediate” subsets expressed a homolog of Blimp1 found in T cells, which directly represses *S1PR1* and *KLF2*, two tissue egress-promoting genes. In contrast, cell proliferation-related genes such as *MKI67* and *STMN1* were highly expressed in the “IC-high” group. Overall, the “IC-high” cluster exhibited the strongest cytotoxicity and proliferative features, whereas the “IC-low” cluster had the lowest expression of IC and cytotoxicity-related molecules. They concluded that the CD103+ CD8+ T cell population may be an important immunotherapeutic target EC, and targeting this cell population with combined immunosuppressive therapy might improve the efficacy of immunotherapy for EC.

Regarding the markers used for the identification of T cell subclusters, there is high consistency across studies, as depicted in see **Supplementary Table 7**. For instance, markers such as *CTLA4, PDCD1*, and *HAVCR2* are consistently utilized for identifying exhausted T cells, while *FOXP3* and *IL7R* are commonly employed for Tregs and naive T cells, respectively. Each study also identifies specific T cell populations, such as TRM cells (35) or activated (pro-memory) CD8+ T cells (28).

##### 2.5.1.2 B cells

The role of B cells in cancer immune response, particularly when organized into tertiary lymphoid structures (TLS), seems to be important. Horeweg et al. (31) investigated the involvement of B lymphocytes and TLS in 6 cases of EC. They identified a total of 1,501 naive B lymphocytes, cyclic/germinal center B lymphocytes, and antibody-secreting cells. Analysis of differential gene expression revealed the association of TLS with the overexpression of *L1CAM*, where mature TLS expressed this gene independently of *L1CAM* expression in the tumor. The study observed that TLS expressing *L1CAM* are more prevalent in EC cases with deficient mismatch repair and mutations in polymerase-epsilon. Furthermore, this research demonstrated a strong and favorable prognostic impact of TLS. Their data suggest a fundamental role of TLS in the prognosis of EC patients, establishing *L1CAM* as a simple biomarker.

#### 2.5.2 The heterogeneity of myeloid cells

Wu et al. (30) conducted a comprehensive analysis of macrophage subsets in the EEC of the Regner et al. (29) study and in normal endometrium, revealing distinct functions and intercellular communication patterns within the TME. Three macrophage subsets were identified and two of them exhibited a tissue-specific distribution (cancer or normal endometrium) (see **Supplementary Table 7**; **Figure 6**). Macrophages1 (CXCL8^hi^IL1B^hi^), which they speculated may be SPP1+ TAMs, were predominantly present in EC samples, expressing cytokines associated with tumor cell proliferation, invasion, and metastasis signaling pathways. Macrophages3 (C3^hi^IL1B^lo^), which were similar to the previously reported tissue-resident LYVE1+ macrophages, were mainly found in normal endometrium and played a role in antigen presentation, with different signaling pathways activated compared to Macrophage1. Surprisingly, Macrophages2 (C1Q^hi^IL1B^lo^), which may be C1QC + TAMs, were present in both EC and normal endometrial tissue, exhibiting completely different functions in each context. In EC environment, they performed various cancer-related functions and shared receptor–ligand pairs with CXCL8^hi^IL1B^hi^ macrophages, while in normal endometrium, its roles were related to metabolism and autoimmune diseases and shared pairs with C3^hi^IL1B^lo^ macrophages.

In terms of intercellular interactions, CXCL8^hi^IL1B^hi^ macrophages demonstrated strong crosstalk with fibroblasts, smooth muscle cells, endothelial cells, proliferating T cells, and tumor epithelial cells in the TME. Notably, secretory ligands *SPP1* and *NAMPT* in CXCL8^hi^IL1B^hi^ macrophages played a role in tumorogenesis by sending signals to receptors on stromal and tumor cells. Additionally, CXCL8^hi^IL1B^hi^ macrophages communicated with epithelial cells through *VEGFA*, contributing to tumor vascular function. On the other hand, C3^hi^IL1B^lo^ macrophages exhibited intercellular communication with normal epithelial and stromal cells, as well as other immune cells, suggesting a role in maintaining immune balance in normal endometrial tissue. Finally, Wu et al. (30) constructed a gene expression signature in macrophages using the genes *SLC8A1*, *TXN*, *ANXA5*, *CST3, CD74*, and *NANS*, which had the ability to predict the prognosis in EC patients.

Guo et al. (28) also distinguished three groups of macrophages that behaved according to a continuous activation model, starting with OLR1+ macrophages, followed by C1QC+ macrophages, and concluding with MARCO+ macrophages. In the study by Wu et al. (30), they speculated that the C1Q^hi^IL1B^lo^ macrophage population could correspond to the C1QC+ population. In both studies, this population corresponded to intermediate macrophages and was found in both EC and normal endometrial tissue. Furthermore, in Guo et al. (28) study, macrophages were significantly enriched in the tumor compared to normal tissue (84.5% vs. 36.2%), conversely Ren et al. (33) reported no significant differences, and tumor infiltrating macrophages were associated with increased overall survival.

Additionally, when characterizing macrophages in terms of pro-inflammatory and anti-inflammatory signatures, a negative correlation was observed between macrophage activation and the enrichment of pro-inflammatory factors, while no association was found with the level of anti-inflammatory factors. These findings suggest that macrophage activation in the tumor microenvironment does not adhere to the polarization model, where M1 and M2 activation states are considered mutually exclusive discrete states. Consistent with this finding, the study by Ren et al. (33) challenged the model of macrophage polarization, as they found that genes associated with both M1 (such as *IL1A* and *IL1B*) and M2 (such as *CD163* and *IL10*) phenotypes were frequently co-expressed within the same macrophage populations.

Ren et al. (33) classified macrophages into three groups: FCN1+ macrophages, SPP1+ macrophages, and cycling macrophages. They observed no significant change in the proportion of these subtypes among macrophages from normal, AEH, and EEC samples. GO analysis of these macrophage subtypes revealed that FCN1+ macrophages were associated with the positive regulation of cytokine production and cellular response, while SPP1+ macrophages were linked to the positive regulation of lymphocyte activation. Additionally, the cycling subtype of macrophages was enriched in cell cycle-related pathways. (See **Figure 6** and see **Supplementary Table 7**).

In the study by Wu et al. (30), it was speculated that the population of CXCL8^hi^IL1B^hi^ macrophages may correspond to SPP1+ TAMs, which constituted the predominant macrophage population in Ren et al.’s study (33). However, in Wu et al. (30), this population was predominantly present in EC samples, expressing cytokines associated with tumor cell proliferation, invasion, and metastasis signaling pathways. In contrast, Ren et al. (33) found no significant differences in the proportions of these macrophages between tumor and healthy tissue, where they were associated with the positive regulation of cytokine production and cellular response.

Unlike macrophages, the remaining myeloid cells analyzed in Guo et al.’s study (28), including monocytes, dendritic cells (DCs), and mast cells, showed a higher percentage in normal tissue compared to tumor tissue. Two subtypes of monocytes were subjected to further analysis: population 1, characterized by CD14+ S100A12+, was transcriptionally similar to “classical” monocytes, while population 2, defined by FCGR3A+, resembled “nonclassical” monocytes. DCs were further categorized into cDC1 (cross-presenting dendritic cells; CLEC9A+ and XCR1+), cDC2 (CD1C+), and plasmacytoid DC (pDC; LILRA4+ and IL3RA+). This detailed analysis reveals distinct transcriptional profiles and subtypes within the myeloid cell populations, highlighting their differential distribution between normal and tumor tissues. (See **Supplementary Table 7**; **Figure 6**).

## 3 Discussion and conclusions

The development of scRNA-seq techniques has provided novel insights into both healthy (36, 38, 39) and tumor endometrium (27–32, 36, 38, 39). ScRNA-seq analysis is revolutionizing our understanding of EC biology by allowing the identification and characterization of different cellular populations. This technique sheds light on tumor heterogeneity and unravels the intricate communication networks between various cell types, holding immense potential for advancing our knowledge of EC pathogenesis, identification of new biomarkers and the development of targeted therapies.

While this technology has made significant advancements in the study of the endometrium, certain limitations persist. Among the various studies included in this review, discrepancies exist in methodological aspects. For instance, variations were observed in the dissociation techniques employed to isolate individual cells from tissue samples. None of these authors used an automatic dissociator; discrepancies range from the type of medium used for enzymatic digestion, the type of enzymes for digestion, with some of those used in these studies being collagenase I, II, IV, V hyaluronidase, the percentage at which they are used also varies, in addition to digestion times ranging from 15 minutes at 37°C to overnight at room temperature. Some authors complement the initial digestion with mechanical trituration through the use of a 1 ml syringe plunger (28) or perform a second dissociation with trypsin when deemed necessary (29).

Additionally, differences in single cell technology or sequencing platform selected and sequencing parameters contributed to methodological disparities. In most studies reviewed here, the 10X Genomics technology was used (27–30, 32–35), with the exception of the study Horeweg et al. (31), which utilized SMART-seq technology (see **Supplementary Table 4**).

Furthermore, the computational analysis phase introduced additional diversity, with variations in the choice of reference genome, normalization methods, filtering criteria, doublet elimination, and batch effect correction to process the sequencing data. All studies included in this review specified the use of the GRCh38 reference genome, except for Gou et al. (28) and Jiang et al. (35). **Supplementary Table 3** illustrates the differences in gene expression, UMI counts, or mitochondrial read fraction filtering criteria used in each study. For instance, Gou et al. (28) employed the strictest filter, eliminating all cells with a mitochondrial fraction > 5%. Following the filtering process, only two of the studies specified the elimination of doublets (27, 29), and four corrected for batch effects that may arise when merging samples (31–34). This correction could potentially remove some patient-specific heterogeneity. (See **Supplementary Table 4**).

Cell annotation, which is one of the main challenges, also varied, ranging from manual inspection using marker genes to the application of automated tools like singleR for cell type identification, and there were also differences in the number of PCs and clustering resolution. In **Supplementary Table 4**, it can be observed that not all studies used a common annotation method, which may affect the identified cell populations. Another methodological challenge in scRNAseq studies is distinguishing malignant epithelial cells populations from normal cells. In 3 of the studies, the detection of tumor cells was carried out through the analysis of CNVs. In the studies by Ren et al. (33) and Yu et al. (27), normal epithelial cells were selected as a reference, while in the article by Gou et al. (28), stromal cells were taken as a reference and assigned as “normal.” On the other hand, Regner et al. (29) and Yu et al. (32) detected tumor cells based on the expression levels of the approved biomarkers *MUC16/CA125*. Notably, Regner et al. (29), despite using the CNV detection technique in addition to biomarker genes to identify tumor cells in ovarian cancer samples, did not apply it to endometrial samples, arguing that CNVs are not relevant in EC. (See **Supplementary Table 4**)

A major limitation, which is partially due to the current complexity and cost of the technique, is the low number of cases so far analyzed. In fact, this review includes only 29 tumors, which are not representative of the complete spectrum of EC. The predominant histological type was consistent across all EC studies, with endometrioid being the most common (*n*=19) (27–30, 32–34), followed by serous (*n*=1) (29), with 9 cases where the histological type was not specified (31, 35). Molecular subtype information, which is crucial for prognosis and immune response assessment, was specified in only 12 cases. Additionally, age variability (ranging from 42 to 82 years old) could potentially act as a confounding factor in the analyses. (See **Supplementary Table 2**)

Additionally, the number of cells analyzed per case varied across studies (ranging from 2,500-8,500 cells/case in tumor tissue), which may impact the ability to identify and characterize different cellular subpopulations present in the sample and affect the resolution of cellular heterogeneity (see **Supplementary Table 1**). Furthermore, the percentage of CAFs found among samples of the same histological type, EEC, also differed between studies. Wu et al. (30) identified a significant fibroblast population, which was the largest among the identified cell types in tumor samples. In contrast, Guo et al. (28) generally observed low fractions of fibroblasts (<5%) in the majority of cases. This discrepancy was attributed to potential inefficiencies during the tissue dissociation process, as fibroblasts and endothelial cells are more embedded in the extracellular matrix and the basement membrane than other cell types. However, another plausible explanation is intertumor heterogeneity. It is well-known that the percentage of tumor and stromal cells is highly variable among tumor samples and for this reason is probably that some of the identified cellular subgroups were highly patient-specific.

In conclusion, it is crucial to continue conducting scRNA-seq analysis in EC cases. The methodological disparities discussed here underscore the importance of carefully considering and standardizing experimental protocols in scRNA-Seq studies to ensure robust and comparable results. In addition, future studies should increase the number of cases studied and include the various histological and molecular types. Particularly, it would be highly beneficial to include aggressive types of EC, such as serous carcinomas, clear cell carcinomas, carcinosarcomas and p53 abnormal carcinomas, as there is currently a gap in the literature. It is also important to consider that the TME differs between subtypes of EC, such as in POLE and MMRd tumors. For instance, in other tumors, mutations in beta-catenin have been suggested to be associated with an immunosuppressive environment (40, 41). Furthermore, including more samples from early stages of EC such as AEH, and analyzing multiple samples from the same tumor at different stages of progression (e.g., primary tumors and metastases) or after specific therapies, will enhance our understanding of EC.
